# Why Do Males in Scotland Die Younger than Those in England? Evidence from Three Prospective Cohort Studies

**DOI:** 10.1371/journal.pone.0038860

**Published:** 2012-07-11

**Authors:** Gerry McCartney, Martin Shipley, Carole Hart, George Davey-Smith, Mika Kivimäki, David Walsh, Graham C. Watt, G. David Batty

**Affiliations:** 1 Public Health Science Directorate, NHS Health Scotland, Glasgow, Scotland; 2 Department of Epidemiology and Public Health, University College London, London, United Kingdom; 3 Institute of Health and Wellbeing, University of Glasgow, Glasgow, Scotland; 4 MRC Centre for Causal Analyses in Translational Epidemiology, School of Social and Community Medicine, University of Bristol, Bristol, United Kingdom; 5 Glasgow Centre for Population Health, Glasgow, Scotland; 6 Section of General Practice and Primary Care, Institute of Health and Wellbeing, University of Glasgow, Glasgow, Scotland; 7 Centre for Cognitive Ageing and Cognitive Epidemiology, Department of Psychology, The University of Edinburgh, Edinburgh, Scotland; Cardiff University, United Kingdom

## Abstract

**Background:**

To examine explanations for the higher rates of male mortality in two Scottish cohorts compared with a cohort in south-east England for which similar data were collected.

**Methodology/Principal Findings:**

We compared three cohort studies which recruited participants in the late 1960s and early 1970s. A total of 13,884 men aged 45–64 years at recruitment in the Whitehall occupational cohort (south-east England), 3,956 men in the Collaborative occupational cohort and 6,813 men in the Renfrew & Paisley population-based study (both central Scotland) were included in analyses of all-cause and cause-specific mortality. All-cause mortality was 25% (age-adjusted hazard ratio 1.25, 95% confidence interval (CI)1.21 to 1.30) and 41% (hazard ratio 1.41 (95% CI 1.36 to 1.45) higher in the Collaborative and Renfrew & Paisley cohorts respectively compared to the Whitehall cohort. The higher mortality rates were substantially attenuated by social class (to 8% and 17% higher respectively), and were effectively eliminated upon the further addition of the other baseline risk factors, such as smoking habit, lung function and pre-existing self-reported morbidity. Despite this, coronary heart disease mortality remained 11% and 16% higher, stroke mortality 45% and 37% higher, mortality from accidents and suicide 51% and 70% higher, and alcohol-related mortality 46% and 73% higher in the Collaborative and Renfrew & Paisley cohorts respectively compared with the Whitehall cohort in the fully adjusted model.

**Conclusions/Significance:**

The higher all-cause, respiratory, and lung cancer male mortality in the Scottish cohorts was almost entirely explained by social class differences and higher prevalence of known risk factors, but reasons for the excess mortality from stroke, alcohol-related causes, accidents and suicide remained unknown.

## Introduction

Life expectancy in Scotland was comparable to the rest of western Europe until around 1950 [1]. From 1950 onwards, all-cause mortality rates in Scotland have improved more slowly than elsewhere in western Europe and diverged from those in England & Wales [2], [3]. Between 1950 and 1980, the higher mortality in Scotland was primarily driven by deaths due to cardiovascular disease, stroke, respiratory disease and cancer amongst men and women of middle age. It is believed that much of this higher mortality rate may be accounted for by greater poverty and the consequences of this [4], but there are no analyses of mortality and deprivation comparing Scotland and England prior to the creation of postcodes (zip-codes) in the 1970s.

From the 1980s onwards the pattern of mortality changed in Scotland. Mortality rates related to alcohol, illicit drugs, suicide and violence increased in young men and young women [2], [3]. In Scotland overall, and in west central Scotland in particular, this meant a rise in male mortality for young adult males in absolute terms [5], [6]. Although the mortality rates for cardiovascular disease, stroke and cancer in middle age improved from this time, they remained high relative to England & Wales and the rest of western Europe. In 1981, all-cause mortality in Scotland was 12% higher than in England & Wales, rising to 15% higher by 2001. However, the proportion of this rising excess explained by the Carstairs deprivation index (a measure of area deprivation derived from Census data on social class, overcrowding, car ownership and unemployment) [7] actually declined from 62% in 1981 to 47% in 2001.

The increasing proportion of the mortality gap between Scotland and England unexplained by deprivation has been termed the ‘Scottish Effect’ [8]. The phenomenon of higher mortality not entirely explained by deprivation has also been confirmed at city level, where premature mortality in Glasgow is seen to be 30% higher than in the equally deprived English cities of Liverpool and Manchester (as approximated by income-deprivation prevalence in small areas) [9], and in mortality from ischaemic heart disease in Scotland compared to England using individual data [10]. The suggestion that something additional to deprivation is impacting on health in Scotland is also supported by the rising premature mortality in Scotland’s persistently deprived areas in contrast to the declining trends seen in England [11].

The changing mortality pattern in Scotland emerged approximately one decade after the recruitment of participants into three cohorts in south-east England (the Whitehall I study of male London based civil servants between 1967 and 1970 [12]) and central Scotland (the Collaborative Study of employees from a wide variety of workplaces around Glasgow, Clydebank and Grangemouth screened between 1970 and 1973 [13]; and the general population Renfrew & Paisley study carried out between 1972 and 1976 [14]). The selected participants in the two occupational cohorts were subject to the ‘healthy worker effect’ since they included only those who were in employment. The methods used and the data collected in the Whitehall cohort were very similar to that in the Scottish cohort studies.

The three cohort studies therefore provide the opportunity to look at the higher mortality in Scotland compared to England through a different lens, by examining the extent to which any mortality differences between the cohorts can be explained by social class and biological characteristics and behavioural risk factors.

## Methods

### The Studies

In the Whitehall study, 18,403 men aged 40–64 years were examined between 1967 and 1969 [12]. Of this total, 15,395 of the men were aged 45–64 years (the age group recruited in the Renfrew & Paisley cohort). The Collaborative cohort of men and women was recruited from 27 workplaces in Glasgow, Grangemouth, and Clydebank (in central Scotland) between 1970 and 1973 [13]. Response rates were available for the workplaces from which 87% of the sample was recruited. For these sites 70% of those invited completed the questionnaire and attended for examination. The achieved sample included 6,022 men, of whom 4,021 were aged 45–64 years. The Renfrew & Paisley general population study was carried out between 1972 and 1976. The sampling frame was residents of the towns of Renfrew & Paisley (in central Scotland) aged 45–64 years, and a 78% response was achieved. Full details of the study methodology have been reported previously [14]. A total of 7,049 men were included in the study. Due to their geographical locations, there were 26 men who took part in both the Collaborative and Renfrew & Paisley studies. To ensure they were only included once in the combined analysis, their records from the Renfrew & Paisley study were not used, leaving 7,023 men.

The questionnaire and examination procedures were similar in all three studies as they had been developed from an earlier occupational health survey carried out in the west of Scotland between 1964 and 1967 and there was close collaboration between the cohort founders Victor Hawthorne and Geoffrey Rose [15]. Cohort participants completed a questionnaire to collect data on demographics, occupation and smoking habit. In the Scottish cohorts, occupation was coded and assigned to social class using the Registrar General Classification. In the Whitehall study, civil service employment grade was categorised as administrative, professional or executive, clerical, and “other grades” (men in ‘messenger’ and other unskilled manual jobs) and matched to the Registrar General Classification of social class [16]. Smoking was categorised according to cigarette, pipe or cigar use (as “never smoker”, “ex-cigarette smoker”, “current cigarette smoker” or “current pipe or cigar smoker”). In addition, the number of cigarettes smoked per day was recorded and controlled for in the smoking adjusted analyses. The Rose angina questionnaire [17] and the Medical Research Council respiratory questionnaire [18] were also included. As part of these questionnaires persistent phlegm was defined as: usually bringing up phlegm from the chest first thing in the morning on most days for three months during winter each year. “Infective phlegm” was defined as: usually bringing up phlegm from the chest first thing in the morning in winter and having had a period of increased cough and phlegm lasting for three weeks or more in the previous three years. Breathlessness was defined as a positive response to the question: “Do you get short of breath walking with people of your own age on level ground?” and bronchitis as having persistent or “infective” phlegm and being breathless. Angina was considered present if chest pain or discomfort when walking uphill or hurrying was cited in the sternum or the left chest and arm; caused the subject to stop or slow down; went away when the subject stopped or slowed down; and went away in 10 minutes or less.

The examination measurements included height, weight, blood pressure, lung function, a six lead electrocardiogram and plasma cholesterol concentration in all three cohorts. Heights were measured with shoes on in the Whitehall study, so a deduction of 2.54 cm (1 inch) in height was made for each participant to improve comparability with the other cohorts (a sensitivity analyses was also conducted using a deduction of 1.27 cm, or ½ inch). In the Whitehall study, the cholesterol measures taken later in the baseline data collection period were systematically lower than those at earlier times and may have been adversely affected by a change in the concentration of the laboratory standard over time. The cholesterol values were therefore adjusted to their predicted values as if they were taken at the start of the baseline data collection period (which had the effect of increasing the mean cholesterol in the Whitehall cohort from 5.12 to 5.69).

The electrocardiogram was coded according to the Minnesota system [19] and was regarded as positive for ischaemia if Q/QS items (codes 1.1–3), ST/T items (codes 4.1-4 or 5.1-3), or left bundle branch block (code 7.1) were present. Forced expiratory volume in 1 second (FEV_1_) was also recorded. The FEV ratio was calculated for each individual as the ratio of measured FEV_1_ divided by predicted FEV calculated from the subjects’ age and height based on an equation derived in those men free of respiratory symptoms in the Renfrew & Paisley study.

Records were traced and flagged at the National Health Service Central Registry. Death certificates coded according to the eighth, ninth or tenth revision of the International Classification of Diseases (ICD), were obtained. Mortality was classified as being due to coronary heart disease (ICD8/9: 410–414; ICD10: I20–I25), stroke (ICD8/9: 430–438; ICD10: I60-I69), respiratory disease (ICD8/9: 460–519; ICD10: J00–J99), lung cancer (ICD8/9: 162; ICD10: C33–C34), accidents and suicide (ICD8/9: E800–E999; ICD10: S00-Y98) or alcohol-related deaths (ICD8/9: 141, 143–6, 148–9, 150, 155, 161, 291, 303, 571 or E800–E999; ICD10: C01–C06, C10, C13–C15, C22, C32, F10, K70, K74.6 or S00-Y98). Mortality follow-up was until 31^st^ December 2008.

### Statistical Analysis

We excluded from the three studies a total of 1,775 (6.8%) men with missing values for any of the covariates and those who were not followed up for mortality. This resulted in an analytic sample of 24,653 men (13,884 Whitehall, 3,956 Collaborative and 6,813 Renfrew & Paisley). The prevalence of baseline characteristics in the three studies was adjusted for age (5-year age groups) using direct standardisation with the combined population as the standard. Differences in prevalence between the studies were tested for significance using the Mantel-Haenzsel test. For continuous variables, least-squares means were used to present the age-adjusted means and the significance of the study group variable was used to test for heterogeneity.

Mortality rates, by follow-up period and overall, were calculated using person years at risk and were standardised for age at entry as above. Hazard ratios and 95% confidence intervals for mortality in the Scottish cohorts compared to the Whitehall study were computed using Cox’s proportional hazards regression model with follow up time as the time scale. Initially, models were adjusted for age and each of the potential explanatory factors separately. Subsequently, multiply adjusted models controlled for all factors both including and excluding social class. An analysis stratified by social class was also performed adjusting for all other factors. Adjustment for BMI in these models was achieved by including both linear and quadratic BMI terms [20]. All other continuous measures were controlled for by including a single linear term. To adjust for smoking we used three indicator variables to compare ex-smokers, pipe/cigar smokers and current smokers with never smokers (with one indicator variable for each comparison) and fitted a term that adjusted for the numbers of cigarettes smoked per day in the current smokers. Proportional hazard models were also fitted within each of the social class groups to examine the magnitude of the hazard ratios and to assess the effect of controlling for the explanatory factors within each group. Further models were fitted to compare the hazard ratios and the effect of the adjustments by stratifying the follow-up into periods 0–9 years, 10–19 years and ≥20 years.

## Results

The distribution of baseline characteristics of the men in the three cohort studies are presented in table 1. As expected, the Whitehall cohort has a much higher proportion of individuals in social classes I and II (72.7%) compared to the Collaborative study (30.4%) and Renfrew & Paisley study (19.3%). The high proportion of people in social class IIIM, IV and V in the Collaborative (52.8%) and Renfrew & Paisley (69%) cohorts is unusual for a study of this type, but reflects the social class make-up of the communities from which the cohorts are drawn. Cholesterol levels were similar in the Collaborative and Renfrew & Paisley studies, and much lower in the Whitehall study. Blood pressure was similar in the Whitehall and Collaborative studies, and higher in the Renfrew & Paisley study. BMI was highest in the Renfrew & Paisley study and lowest in the Collaborative study. Men in the Whitehall study were the tallest and had the best FEV_1_ and men in the Renfrew & Paisley study the shortest with the worst FEV_1_. The largest baseline differences in the questionnaire measures relate to smoking (where there is a higher proportion of ex-smokers in the Whitehall study and lower proportion of current smokers) and self-reported morbidity (for cardiorespiratory symptoms and previous diagnoses).

**Table 1:** Proportions and means+ for established risk factors by study.

	Whitehall(n = 13,884)	Collaborative(n = 3,956)	Renfrew & Paisley(n = 6,813)
Age (years)	53.6	52.2	54.6
Social class (%)			
I, II	72.7	30.4	19.3
III NM	16.9	16.9	11.6
III M, IV, V	10.4	52.8	69.0
Plasma cholesterol (mmol/l)	5.69	5.88	5.86
Systolic blood pressure (mm Hg)	137.4	137.5	147.6
Diastolic blood pressure (mm Hg)	85.0	84.9	85.8
Body Mass Index (kg m^−2^)	25.6	25.2	25.9
Height (cm)	172.9	171.9	169.7
Smoking status (%):			
Never	17.0	15.0	16.8
Ex	37.3	27.3	24.3
Current pipe or cigar smoker	3.5	2.5	1.9
Current cigarette smoker	42.2	55.2	57.0
Cigarettes per day (smokers only)	16.1	18.8	20.5
Forced Expiratory Volume in 1 second (FEV_1_)			
Mean	3.07	2.81	2.56
Ratio to that predicted*	1.04	0.96	0.90
Angina (%)	5.3	7.7	8.9
Possible MI (%)	7.0	7.8	9.2
ECG abnormalities (%)	7.2	7.3	9.8
Respiratory symptoms (%)			
No winter phlegm	76.4	69.8	63.5
Persistent phlegm	16.2	19.4	21.4
Infective phlegm	7.4	10.8	15.1
Breathlessness (%)	6.1	7.2	12.9
MRC Chronic bronchitis (%)	3.2	4.3	8.7

+ Prevalences and means are adjusted for age (age is unadjusted). All measures show significant (p<0.001) heterogeneity between the studies.

* FEV ratio is the ratio of measured FEV divided by predicted FEV calculated from subjects’ age and height based on an equation derived in those men free of respiratory symptoms in the Renfrew & Paisley study.

Table 2 gives the number of deaths and the age standardised mortality rates in each cohort by cause of death. The largest number of deaths was due to coronary heart disease (CHD), followed by respiratory causes, stroke and lung cancer. The mortality rates increase across the follow-up periods since the men are older in the later periods of follow-up and are consistently higher in the Renfrew & Paisley than the Collaborative cohort and the Whitehall cohort (at 41.6, 38.7 and 35.8 deaths per 1,000 person years respectively). The relative risk of mortality after age-adjustment was higher in both Scottish cohorts than in the Whitehall study for each specific cause and for all causes (Table 3). All-cause mortality was 25% and 41% higher, CHD mortality 32% and 41% higher, stroke mortality 55% and 73% higher, respiratory mortality 5% and 17% higher, lung cancer mortality 65% and 98% higher, mortality from accidents and suicide was 77% and 100% higher, and alcohol-related mortality 73% and 128% higher in the Collaborative and Renfrew & Paisley cohorts respectively as compared to the Whitehall cohort. The higher mortality rates were substantially attenuated with the addition of socio-economic position to the model, but remained higher for all-causes (8% and 17%), CHD (17% and 22%), stroke (45% and 60%), lung cancer (16% and 30%), accidents and suicide (56% and 70%), and alcohol-related causes (47% and 85%). The addition of single biological or behavioural factors in addition to age in the model (including smoking, FEV_1_, cardio-respiratory symptoms or history, height, blood pressure and cholesterol) were unable to explain as much of the higher all-cause (or any of the specific causes) mortality in the Scottish cohorts as socio-economic position (with the exceptions of FEV_1_ for CHD mortality and blood pressure for stroke mortality, in the Renfrew & Paisley study).

**Table 2:** Age adjusted mortality rates by study and time of follow-up.

Follow-up period								
	0–9 years		10–19 years		≥20 years		Total	
	Deaths	Rate* (SE)	Deaths	Rate* (SE)	Deaths	Rate* (SE)	Deaths	Rate* (SE)
All causes								
Whitehall	1,557	12.1 (0.3)	2,793	28.0 (0.5)	7,336	84.4 (1.2)	11,686	35.8 (0.3)
Collaborative	527	16.1 (0.8)	897	36.0 (1.5)	1,818	84.4 (1.0)	3,242	38.7 (0.8)
Renfrew & Paisley	1,327	19.9 (0.6)	1,927	43.4 (1.6)	2,457	90.6 (1.1)	5,711	41.6 (0.6)
CHD								
Whitehall	665	5.2 (0.2)	969	9.6 (0.3)	1,785	20.2 (0.6)	3,419	10.6 (0.2)
Collaborative	235	7.0 (0.5)	346	13.4 (0.9)	464	21.0 (1.4)	1,045	12.4 (0.5)
Renfrew & Paisley	554	8.4 (0.4)	681	15.4 (0.6)	596	21.6 (1.0)	1,831	13.4 (0.3)
Stroke								
Whitehall	84	0.7 (0.1)	218	2.2 (0.2)	783	9.4 (0.4)	1,085	3.4 (0.1)
Collaborative	41	1.4 (0.3)	81	4.1 (0.6)	229	10.8 (1.0)	351	4.5 (0.3)
Renfrew & Paisley	94	1.4 (0.1)	192	4.4 (0.3)	334	13.1 (0.8)	620	4.5 (0.2)
Respiratory diseases								
Whitehall	100	0.8 (0.1)	284	3.0 (0.2)	1,171	15.0 (0.5)	1,555	4.9 (0.1)
Collaborative	23	0.8 (0.2)	74	3.2 (0.5)	238	13.4 (1.3)	335	4.3 (0.3)
Renfrew & Paisley	104	1.5 (0.2)	170	3.9 (0.3)	324	12.9 (0.9)	598	4.4 (0.2)
Lung Cancer								
Whitehall	180	1.4 (0.1)	264	2.7 (0.2)	344	3.7 (0.2)	788	2.5 (0.1)
Collaborative	69	2.1 (0.3)	90	3.6 (0.5)	148	5.3 (0.5)	307	3.5 (0.2)
Renfrew & Paisley	185	2.8 (0.2)	247	5.6 (0.4)	182	6.2 (0.5)	614	4.5 (0.2)
Accidents and suicide								
Whitehall	178	1.4 (0.1)	137	1.3 (0.1)	88	1.0 (0.1)	178	0.5 (0.1)
Collaborative	68	1.7 (0.2)	51	1.7 (0.3)	33	1.3 (0.3)	68	0.7 (0.1)
Renfrew & Paisley	117	1.8 (0.2)	81	1.8 (0.2)	49	1.9 (0.3)	117	0.9 (0.1)
Alcohol-related								
Whitehall	390	3.0 (0.2)	324	3.0 (0.2)	219	2.4 (0.2)	390	1.2 (0.1)
Collaborative	156	3.9 (0.3)	122	3.9 (0.4)	77	3.0 (0.5)	156	1.6 (0.2)
Renfrew & Paisley	298	4.8 (0.3)	215	4.8 (0.3)	127	4.3 (0.4)	298	2.2 (0.1)

* Rates are given as the number of deaths per 1,000 person-years at risk.

After adjusting for all risk factors except social class, all-cause mortality remained 9% and 7% higher, CHD mortality remained 16% and 1% higher, stroke mortality 44% and 36% higher, lung cancer mortality 24% and 38%, mortality due to accidents and suicide 57% and 78% higher and alcohol-related mortality 56% and 88% higher in the Collaborative and Renfrew & Paisley cohorts respectively compared to the Whitehall cohort. Respiratory mortality adjusts to be lower in the Scottish cohorts (Table 3), despite it being higher before adjustment for the earliest time period after baseline data collection (Table 2).

**Table 3:** Hazard ratios for mortality in the Scottish cohorts compared to the Whitehall Study^+^ (reference) with successive adjustment for potential explanatory factors.

Adjusted for	All-cause mortality		CHD mortality		Stroke mortality		Respiratory mortality		Lung cancer mortality		Mortality due to accidents and suicide		Alcohol-related mortality	
	Collaborative	Renfrew &Paisley	Collaborative	Renfrew &Paisley	Collaborative	Renfrew &Paisley	Collaborative	Renfrew &Paisley	Collaborative	Renfrew &Paisley	Collaborative	Renfrew &Paisley	Collaborative	Renfrew &Paisley
	HR (95% CI)	HR (95% CI)	HR (95% CI)	HR (95% CI)	HR (95% CI)	HR (95% CI)	HR (95% CI)	HR (95% CI)	HR (95% CI)	HR (95% CI)	HR (95% CI)	HR (95% CI)	HR (95% CI)	HR (95% CI)
Age	1.25 (1.21,1.30)	1.41 (1.36,1.45)	1.32 (1.23,1.41)	1.41 (1.33,1.50)	1.55 (1.37,1.75)	1.73 (1.57,1.92)	1.05 (0.93,1.18)	1.17 (1.07,1.29)	1.65(1.45,1.89)	1.98 (1.78,2.21)	1.77 (1.33,2.36)	2.00 (1.56,2.55)	1.73 (1.44,2.09)	2.28 (1.95,2.67)
Age, socio-economicposition	1.08 (1.04,1.13)	1.17 (1.12,1.21)	1.17 (1.09,1.26)	1.22 (1.14,1.31)	1.45 (1.27,1.66)	1.60 (1.42,1.80)	0.78 (0.69,0.88)	0.82 (0.73,0.92)	1.16 (1.01,1.35)	1.30 (1.14,1.47)	1.56 (1.14,2.13)	1.70 (1.27,2.27)	1.47 (1.20,1.81)	1.85 (1.53,2.24)
Age, smoking	1.17 (1.13,1.22)	1.30 (1.26,1.34)	1.25 (1.17,1.34)	1.33 (1.26,1.42)	1.48 (1.31,1.67)	1.63 (1.47,1.81)	0.94 (0.83,1.06)	1.03 (0.93,1.14)	1.34 (1.17,1.53)	1.55 (1.39,1.73)	1.66 (1.24,2.21)	1.86 (1.45,2.38)	1.62 (1.34,1.96)	2.09 (1.78,2.45)
Age, FEV_1_	1.14 (1.10,1.19)	1.19 (1.15,1.23)	1.22 (1.13,1.30)	1.22 (1.15,1.30)	1.46 (1.29,1.65)	1.56 (1.40,1.73)	0.81 (0.72,0.91)	0.76 (0.69,0.84)	1.42 (1.24,1.62)	1.52 (1.36,1.70)	1.64 (1.23,2.19)	1.73 (1.34,2.23)	1.59 (1.32,1.93)	1.96 (1.66,2.30)
Age, cardio-respiratorysymptomsor history*	1.22 (1.18,1.27)	1.31 (1.27,1.35)	1.29 (1.20,1.38)	1.30 (1.23,1.38)	1.54 (1.36,1.74)	1.67 (1.50,1.85)	0.98 (0.87,1.11)	0.99 (0.90,1.10)	1.57 (1.37,1.79)	1.79 (1.60,1.99)	1.72 (1.29,2.29)	1.85 (1.45,2.37)	1.70 (1.40,2.05)	2.13 (1.82,2.49)
Age, height	1.25 (1.20,1.30)	1.37 (1.33,1.42)	1.30 (1.21,1.40)	1.35 (1.28,1.44)	1.53 (1.36,1.73)	1.67 (1.51,1.86)	1.03 (0.92,1.16)	1.12 (1.01,1.23)	1.65 (1.44,1.88)	1.95 (1.75,2.18)	1.74 (1.31,2.32)	1.90 (1.48,2.43)	1.70 (1.41,2.06)	2.16 (1.84,2.53)
Age, bodymass index	1.25 (1.20,1.30)	1.39 (1.35,1.44)	1.33 (1.24,1.43)	1.38 (1.30,1.46)	1.55 (1.37,1.75)	1.71 (1.54,1.89)	1.01 (0.90,1.14)	1.19 (1.08,1.31)	1.60 (1.40,1.83)	2.02 (1.81,2.25)	1.73 (1.30,2.30)	2.02 (1.58,2.58)	1.72 (1.42,2.07)	2.26 (1.93,2.64)
Age, systolicand diastolicblood pressure	1.26 (1.21,1.31)	1.33 1.29,1.38)	1.34 (1.25,1.43)	1.28 (1.20,1.36)	1.58 (1.40,1.79)	1.56 (1.41,1.73)	1.05 (0.93,1.18)	1.15 (1.04,1.27)	1.66 (1.45,1.89)	1.98 (1.77,2.21)	1.77 (1.33,2.35)	2.15 (1.67,2.77)	1.73 (1.44,2.09)	2.30 (1.95,2.70)
Age, cholesterol	1.24 (1.20,1.29)	1.40 (1.35,1.44)	1.28 (1.19,1.37)	1.38 (1.30,1.46)	1.55 (1.37,1.76)	1.74 (1.57,1.93)	1.07 (0.95,1.21)	1.20 (1.09,1.32)	1.70 (1.49,1.94)	2.04 (1.83,2.27)	1.79 (1.34,2.38)	2.02 (1.58,2.58)	1.77 (1.47,2.14)	2.33 (1.99,2.73)
Multiply± adjustedwithout socio-economic position	1.09 (1.05,1.14)	1.07 (1.03,1.10)	1.16 (1.08,1.25)	1.01 (0.95,1.08)	1.44 (1.28,1.64)	1.36 (1.21,1.52)	0.75 (0.67,0.85)	0.70 (0.63,0.78)	1.24 (1.08,1.42)	1.38 (1.22,1.55)	1.57 (1.18,2.11)	1.78 (1.36,2.33)	1.56 (1.29,1.89)	1.88 (1.58,2.23)
Multiply± adjustedincluding socio-economic position	1.03 (0.98,1.07)	0.99 (0.95,1.03)	1.11 (1.03,1.20)	0.96 (0.89,1.03)	1.45 (1.27,1.66)	1.37 (1.21,1.55)	0.70 (0.61,0.79)	0.65 (0.58,0.73)	1.06 (0.92,1.23)	1.16 (1.01,1.33)	1.51 (1.11,2.07)	1.70 (1.25,2.30)	1.46 (1.19,1.80)	1.73 (1.42,2.11)

+Analyses for all-cause mortality are based on 13,884, 3,956 and 6,813 men in the Whitehall, Collaborative and Renfrew & Paisley studies respectively. For cause-specific mortality the number of men in the analyses are 13,850, 3,948 and 6,792 respectively. The number of deaths is given in Table 2.

* Angina, ECG abnormality, respiratory symptoms or breathlessness.

± Multiply adjusted for :- age, smoking, FEV_1_, cardio-respiratory symptoms or history, height, systolic blood pressure, cholesterol, body mass index.

The fully adjusted model (including social class and all other explanatory factors together) explained almost all of the mortality excess in the Scottish cohorts for all-cause mortality but there remained some unexplained excess for the specific causes. Stroke mortality remained 45% and 37% higher, mortality from accidents and suicide 51% and 70% higher, and alcohol-related mortality 46% and 73% higher in the Collaborative and Renfrew & Paisley cohorts respectively compared with the Whitehall cohort. CHD mortality remained 11% higher in the Collabarative study and lung cancer remained 16% higher in the Renfrew & Paisley study in the fully adjusted model as compared to the Whitehall study. As before, respiratory mortality appeared to be lower in the Scottish cohorts with addition of all of the explanatory factors (Table 3).

Given the markedly different social class composition of the three cohorts, the baseline characteristics and hazard ratios adjusted for the biological and behavioural risk factors are also presented stratified by social class in tables 4 and 5. The differences in baseline risk factors were less marked after stratification. Cholesterol levels were lower in the Whitehall study compared to the Scottish cohorts within each social class strata. Systolic and diastolic blood pressure were lower in each strata in the Whitehall cohort than in the Renfrew & Paisley cohort, but was higher in each strata than the Collaborative cohort with the exception of systolic blood pressure amongst those in strata IIIM, IV & V. Mean height in social classes I & II and IIINM were highest in the Collaborative study and lowest in the Renfrew & Paisley study (but highest in the Whitehall study in social classes IIIM, IV & V). Cigarette smoking was more prevalent in social class I & II in the Scottish cohorts compared to Whitehall, but not in the other social class strata. However, amongst those smokers, there were a greater mean number of cigarettes smoked per person in the Scottish cohorts for each social class strata. FEV_1_ was highest, and infective phlegm least common, in the Whitehall study except amongst those in social class IIINM where the mean FEV_1_ was highest and prevalence of infective phlegm lower, in the Collaborative study. The proportion with angina was higher in the Scottish cohorts but there was no consistent pattern for previous MI or for breathlessness. The prevalence of chronic bronchitis was lowest in the Collaborative study in all social classes.

**Table 4:** Proportions and means+ for established risk factors in the Whitehall, Collaborative and Renfrew & Paisley studies by social class.

	Social classes I & II			Social class III NM			Social classes IIIM, IV, V		
	Whitehall (N = 10114)	Collaborative (N = 1220)	Renfrew & Paisley (N = 1297)	Whitehall (N = 2333)	Collaborative (N = 664)	Renfrew & Paisley (N = 804)	Whitehall(N = 1437)	Collaborative (N = 2072)	Renfrew & Paisley (N = 4712)
Age (years)	52.6	51.8	54.0	55.4	52.5	55.3	57.1	52.3	54.7
Plasma cholesterol (mmol/l)	5.71	6.24	6.01	5.69	6.02	5.94	5.54	5.64	5.80
Systolic blood pressure (mm Hg)	137.2	134.2	146.9	137.7	136.2	148.0	138.9	139.6	147.8
Diastolic blood pressure (mm Hg)	84.9	83.9	85.8	84.9	84.2	85.9	85.7	85.6	85.9
Body Mass Index (kg m^−2^)	25.6	25.2	26.0	25.4	25.2	25.7	25.6	25.3	25.8
Height (cm)	173.6	174.8	171.8	171.4	172.7	171.3	170.7	170.0	168.8
Smoking status (%):									
Never	18.4	18.2	22.7	13.8	16.8	18.5	11.0	12.5	14.9
Ex	40.9	33.5	30.4	30.3	26.8	26.8	24.1	23.9	22.2
Current pipe or cigar	4.0	3.7	2.6	1.8	2.1	1.6	2.5	1.9	1.7
Current cigarettes	36.8	44.6	44.2	54.1	54.3	53.1	62.3	61.7	61.2
Cigarettes per day (smokers only)	16.0	20.0	21.2	16.3	18.0	20.0	16.3	18.5	20.4
Forced Expiratory Volume in 1 second (FEV_1_)									
Mean	3.18	3.10	2.80	2.85	2.89	2.65	2.74	2.63	2.47
Ratio to that predicted*	1.06	1.02	0.96	0.98	0.98	0.91	0.95	0.92	0.88
Angina (%)	5.1	6.0	6.9	5.6	8.2	10.0	5.4	8.6	9.3
Possible MI (%)	6.7	5.6	8.0	6.7	8.9	9.7	9.2	8.6	9.5
ECG abnormalities (%)	6.8	6.1	9.7	7.9	8.9	10.6	9.1	7.4	9.6
Respiratory symptoms (%)									
No winter phlegm	79.4	77.3	72.3	70.4	69.5	70.0	66.2	65.7	60.4
Persistent phlegm	14.6	15.3	19.4	19.5	21.5	19.6	20.8	21.0	22.3
Infective phlegm	6.0	7.4	8.3	10.1	8.7	10.4	13.0	13.3	17.3
Breathlessness (%)	4.8	3.4	7.1	8.4	9.1	11.7	11.6	8.7	14.6
MRC Chronic bronchitis (%)	2.1	1.5	4.3	5.2	5.0	6.2	6.7	5.6	10.3

+ Prevalences and means are adjusted for age (age is unadjusted).

* FEV ratio is the ratio of measured FEV divided by predicted FEV calculated from subjects’ age and height based on an equation derived in those men free of respiratory symptoms in the Renfrew & Paisley study.

**Table 5:** Hazard ratios for all-cause mortality in the Scottish cohorts compared to the Whitehall Study^+^ (reference) separately by social class with successive adjustment for potential explanatory factors.

	Social classes I & II			Social class III NM			Social classes IIIM, IV, V		
	Whitehall	Collaborative	Renfrew & Paisley	Whitehall	Collaborative	Renfrew & Paisley	Whitehall	Collaborative	Renfrew & Paisley
**Number of subjects**	10114	1220	1297	2333	664	804	1437	2072	4712
**Number of deaths**	8251	923	1004	2097	560	681	1338	1759	4026
**Hazard ratio (95% CI) for all –cause mortality adjusted for :**									
**Age**	1.0	1.09 (1.02,1.17)	1.22 (1.14,1.30)	1.0	1.03 (0.93,1.13)	1.11 (1.01,1.21)	1.0	1.06 (0.98,1.14)	1.13 (1.06,1.21)
**Age, smoking**	1.0	1.03 (0.96,1.10)	1.16 (1.08,1.24)	1.0	1.00 (0.91,1.10)	1.09 (1.00,1.20)	1.0	1.07 (1.00,1.16)	1.14 (1.07,1.22)
**Age, FEV_1_**	1.0	1.05 (0.98,1.12)	1.10 (1.02,1.17)	1.0	1.03 (0.94,1.14)	1.05 (0.96,1.14)	1.0	1.01 (0.94,1.09)	1.04 (0.97,1.11)
**Age, cardio-respiratory symptoms or history** *	1.0	1.09 (1.02,1.17)	1.18 (1.10,1.26)	1.0	1.02 (0.92,1.12)	1.07 (0.98,1.17)	1.0	1.04 (0.96,1.12)	1.07 (1.01,1.15)
**Age, height**	1.0	1.09 (1.02,1.17)	1.21 (1.13,1.29)	1.0	1.03 (0.94,1.13)	1.11 (1.01,1.21)	1.0	1.05 (0.97,1.13)	1.11 (1.04,1.18)
**Age, body mass index**	1.0	1.10 (1.03,1.18)	1.21 (1.13,1.29)	1.0	1.02 (0.93,1.13)	1.10 (1.01,1.21)	1.0	1.06 (0.98,1.14)	1.14 (1.07,1.21)
**Age, systolic and diastolic blood pressure**	1.0	1.11 (1.04,1.19)	1.14 (1.07,1.22)	1.0	1.03 (0.94,1.14)	1.04 (0.96,1.14)	1.0	1.06 (0.99,1.14)	1.10 (1.03,1.17)
**Age, cholesterol**	1.0	1.06 (0.99,1.13)	1.20 (1.12,1.28)	1.0	1.01 (0.91,1.11)	1.09 (1.00,1.19)	1.0	1.05 (0.98,1.14)	1.12 (1.05,1.20)
**Multiply** ± **adjusted**	1.0	1.00 (0.94,1.08)	0.99 (0.93,1.06)	1.0	0.96 (0.87,1.06)	0.95 (0.86,1.04)	1.0	1.04 (0.97,1.13)	1.02 (0.95,1.09)

* Angina, ECG abnormality, respiratory symptoms or breathlessness.

± Multiply adjusted for :- age, smoking, FEV1, cardio-respiratory symptoms or history, height, systolic blood pressure, cholesterol, body mass index.

Table 5 shows that there is an excess in all-cause mortality in social class I & II of 9% in the Collaborative and 22% in the Renfrew & Paisley study compared to Whitehall after age adjustment, but the excesses estimated in the other social class strata are not so large or precise (3% and 11% for social class IIINM, and 6% and 13% in social classes IIIM, IV and V, for the Collaborative and Renfrew & Paisley cohorts respectively) and those for the Collaborative study may be due to chance. Adding the other baseline characteristics to the model completely removes the excess mortality in the Scottish cohorts within each social class strata.

Table 6 shows the differences in mortality between the cohorts stratified by the follow-up time. It shows that the hazard ratios in the Scottish cohorts in comparison with Whitehall declined over time. The excesses in the 10–19 yr period are slightly less well explained whereas the smaller excesses in the ≥20 yr period are completed explained (from 1.10 and 1.01 in the first 10 years of follow-up to 0.96 and 0.95 after >20 years of follow-up in the fully adjusted model in the Collaborative and Renfrew & Paisley cohorts respectively).

**Table 6:** Hazard ratios for all-cause mortality in the Scottish cohorts compared to the Whitehall Study^+^ (reference) by time of follow-up.

	Follow-up period 0–9 years			Follow-up period 10–19 years			Follow-up period 20+ years		
	Whitehall	Collaborative	Renfrew &Paisley	Whitehall	Collaborative	Renfrew &Paisley	Whitehall	Collaborative	Renfrew & Paisley
**Number of subjects**	13884	3956	6813	12239	3417	5452	9375	2508	3515
**Number of deaths**	1557	527	1327	2793	897	1927	7336	1818	2457
**Hazard ratio (95% CI) for all –cause mortality adjusted for:**									
**Age**	1.0	1.45 (1.31,1.60)	1.64 (1.53,1.77)	1.0	1.40 (1.30,1.51)	1.56 (1.47,1.65)	1.0	1.15 (1.09,1.21)	1.25 (1.19,1.21)
**Multiply** ± **adjusted without socio-economic position**	1.0	1.20 (1.09,1.33)	1.10 (1.01,1.19)	1.0	1.21 (1.12,1.30)	1.13 (1.07,1.22)	1.0	1.02 (0.97,1.08)	1.01 (0.96,1.07)
**Multiply** ± **adjusted including socio-economic position**	1.0	1.10 (0.99,1.23)	1.01 (0.92,1.11)	1.0	1.13 (1.05,1.23)	1.07 (0.99,1.15)	1.0	0.96 (0.91,1.02)	0.95 (0.89,1.00)

± Multiply adjusted for :- age, smoking, FEV_1_, cardio-respiratory symptoms or history, height, systolic blood pressure, cholesterol, body mass index.

The sensitivity analyses using a smaller height correction for measurement in shoes in the Whitehall cohort (of 1.27 cm as opposed to 2.54 cm) are shown in tables S1 and S2. The impact on the hazard ratios is small and does not change the overall findings.

## Discussion

The aim of the study was to examine explanations for the higher rates of male mortality in Scotland compared to England using the data from three cohort studies. Compared with the Whitehall cohort in south-east England, male mortality rates were 25% higher in the Scottish occupational Collaborative cohort and 41% higher in the Scottish population-based Renfrew & Paisley cohort. Adjustment for socio-economic position explained most of this higher mortality because of the stark differences in social class distribution between the cohorts. These observations were confirmed when we stratified the samples according to SES and period of follow-up. Although all-cause mortality was largely explained, there remained substantial excess mortality from stroke, alcohol-related causes, accidents and suicide which were unexplained by the baseline risk factors. Thus, in these cohorts of middle-aged men recruited during the late 1960s and early 1970s, most of the higher total mortality in the Scottish cohorts can be explained by social class, and almost entirely by combining social class with the higher prevalences of some known risk factors such as the number of cigarettes smoked, FEV_1_ and pre-existing self-reported morbidity. This resonates with the conclusion of an earlier comparison of these cohorts using a much shorter follow-up time [21].

### Strengths and Weaknesses of the Study

This study provides a unique insight into the higher mortality in Scotland and the ‘Scottish Effect’ by using very similar cohort study data from Scotland and England to adjust for baseline differences in characteristics known to influence health outcomes. It therefore provides a complementary analysis to the existing ecological analyses [7], [8] and studies using individual mortality data with a shorter follow-up period [10]. It uses individual-level data and includes several objective biological measures in addition to self-reported behaviours and morbidity.

Two of the cohorts were drawn from workplaces and so may the results not be applicable to the general population. There are very large baseline differences between the Whitehall cohort and the Scottish cohorts relating to the much higher proportion of individuals in social class I and II in Whitehall. Although this is adjusted for in the model, it is possible that there remains confounding due to the very different types of occupations recruited in the studies (such as the higher proportion of manual workers, and the potential for direct occupational exposures [22], in the Collaborative study). We have therefore presented analyses stratified by broad social class categories to reduce the potential for residual confounding. However, there may be other unmeasured aspects of social class and deprivation which are not captured in this measure (for example, area-based deprivation [23]), and the process of combining social class categories in order to provide sufficient precision of hazard ratio estimates, may have under-differentiated the social differences and left residual confounding.

The slightly later period of recruitment in the Scottish cohorts, against a background of population health improvements as was seen in the UK at this time, may have resulted in an underestimate of the mortality difference between the cohorts since a cohort recruited slightly earlier would have been expected to have had higher mortality.

The sensitivity analysis of the height correction in the Whitehall cohort, to allow for measurement with shoes on, did not make a large difference to the adjusted hazard ratios and so is unlikely to endanger the validity of the analysis. The sensitivity analyses which stratified by period of follow-up showed similar patterns of results within each period suggesting that the decline in the hazard ratios between the Scottish and Whitehall cohorts over time is unlikely to have affected the overall results. The limited sample sizes in the analyses stratified by social class make the estimates of the excess imprecise and make it difficult to be certain that the observed higher mortality rates in the Scottish cohorts are not due to chance.

Plasma cholesterol levels are a potentially important explanatory factor in the cohort comparisons because they are, on average, lower in the Whitehall cohort. Unfortunately, the cholesterol measures in the Whitehall study demonstrated a downward trend over time between the first samples taken and later samples, suggesting that there may be a systematic error (towards an underestimate) in the measures related to a change in the laboratory standard. We have therefore applied a correction factor based on the predicted cholesterol level at the start of the analysis period, but this may not have fully eliminated the bias in favour of lower cholesterol in the Whitehall cohort. This issue was discussed in earlier publications of data from this cohort [24], and the possibility of some small residual bias is reinforced by the finding in the British Regional Heart Health study of no difference in plasma cholesterol levels between the Scottish sample towns and English sample towns [25]. Overall, given the small impact of cholesterol on the hazard ratios, it is unlikely that any remaining bias would have a large impact on the overall results.

We were limited by a lack of comparative data on alcohol intake, physical activity and diet in the cohorts. It may not therefore be surprising that alcohol-related deaths and mortality due to accidents and suicide were not explicable across the cohort studies given that the baseline risk factors captured in these cohorts were designed to explore cardiovascular disease aetiology. However, given that the fully adjusted model accounts for all of the excess all-cause mortality in the Scottish cohorts, this does not seem to have been important for all-cause mortality.

There were no further consistent waves of data collection across all three cohorts to allow changes in occupation, socioeconomic circumstances or risk factors over time to be accounted for. Given that changes are likely to be socially patterned (for example, smoking cessation and medical treatment for hypertension are both likely to have been more common amongst social classes I & II, and there was a marked rise in alcohol-related mortality in deprived areas during the 1990s [26] and a large increase in relative poverty in Scotland during the 1980s and 1990s [27]), this could have underestimated the impact of these risk factors.

A generalisation of the findings of this study to the populations of Scotland and England & Wales has to be cautious since the Whitehall and Collaborative studies are occupational cohorts and unlikely to be representative of the population as a whole (because of the ‘healthy-worker’ effect). Furthermore, we did not have comparable data for women to carry out any analyses for females.

### Comparison with Previous Studies

A recent synthesis of the various hypotheses to explain the higher mortality in Scotland noted that there are two distinct time phases in the pattern [28], [29]. The earlier phase from around 1950 to 1980 saw higher mortality rates in Scotland as compared to the rest of western Europe largely due to deaths from cardiovascular disease, stroke, respiratory disease and cancer in middle-aged men and women. Prior to this, Scotland had a similar life expectancy to most other western European countries [1], [3] but with marked inequalities in mortality within the nation. From 1980 onwards the pattern of mortality changed. Causes of death which had thus far been relatively uncommon in Scotland such as liver cirrhosis, illicit drug-related deaths, suicide and violent deaths began to increase amongst a younger group of men and women, in addition to a continuing trend of higher deaths due to cardiovascular disease, stroke, respiratory disease and cancer in middle-aged men and women [2]. Indeed, premature male mortality increased from 1981 (and even more markedly from 1991) in some persistently deprived areas in Glasgow [11].

The cohorts included in this study were recruited in their middle age during the late 1960s and early 1970s. By 1981 (the first year in which we have been able to measure the ‘Scottish Effect’ using Carstairs deprivation index data), the surviving (and therefore healthier) participants in the Whitehall study were aged 52–78 years, in the Collaborative study were aged 53–75 years, and in the Renfrew & Paisley study were aged 50–73 years. Therefore, the Scottish cohorts are more likely to be representative of the populations affected by the earlier divergence in Scottish mortality (between 1950 and 1980) and of the continuing higher mortality amongst elderly men from cardiovascular disease, stroke and cancer than of the divergence in premature mortality occurring after 1980 which affected a younger age cohort. This therefore suggests that, not withstanding the difficulties in generalising from the occupational nature of two of the three cohorts, socio-economic circumstances may be the most important explanatory factor for the higher mortality during this earlier period of mortality divergence between 1950 and 1980, and amongst elderly men from the 1980s onwards.

### Implications of the Study

This study suggests that socio-economic status is the most important explanation for the higher mortality amongst Scottish middle aged men during the 1970s and amongst late middle aged and elderly men in the 1980s and 1990s. This means that the differences in observed male mortality are health inequalities as they arise from the social circumstances in which people live. This paper supports the large existing body of literature which suggests that a redistribution of income and power between socioeconomic groups and geographical areas is likely to be an effective means of reducing health inequalities [30], [31]. Comparisons of more recent and younger cohorts may be able to generate better insights into the more recent divergence in premature mortality between Scotland and England and in determining the causes of the ‘Scottish Effect’ which emerged from 1981 onwards.

### Conclusions

The higher all-cause male mortality in two Scottish cohorts compared to an English cohort all of which recruited participants in the late 1960s and early 1970s was accounted for mostly by differences in social class, and almost entirely by social class and cardiovascular risk factors, but not for some specific causes (stroke, alcohol-related causes, accidents and suicide). This provides further support for policy which redistributes income and power as a means to reducing health inequalities between social classes and geographical areas. The use of cohort studies which recruited younger groups from the 1980s onwards in Scotland and England may be able to shed further light on the causes of the divergent premature mortality trends during this later period.

## Supporting Information

Table S1 **Sensitivity analyses showing hazard ratios for mortality in the Scottish cohorts compared to the Whitehall Study+ (reference) when Whitehall heights are reduced by half an inch (rather than one inch).**
(DOC)

Table S2 **Sensitivity analyses showing hazard ratios for all-cause mortality in the Scottish cohorts compared to the Whitehall Study+ (reference) when Whitehall heights are reduced by half an inch (rather than one inch).**
(DOC)

## References

[1] McCartney G, Walsh D, Whyte B, Collins C (2011). Has Scotland always been the ‘sick man’ of Europe?. European Journal of Public Health.

[2] Whyte B (2007). Scottish mortality in a European context 1950-2000: an analysis of comparative mortality trends.. Edinburgh: Scottish Public Health Observatory.

[3] Leon D, Morton S, Cannegieter S, McKee M (2003). Understanding the health of Scotland’s population in an International context: a review of current approaches, knowledge and recommendations for new research directions.. London: London School of Hygience and Tropical Medicine & Public Health Institute for Scotland.

[4] Shaw M, Davey-Smith G, Dorling D (2005). Health inequalities and New Labour: how the promises compare with real progress.. BMJ.

[5] Walsh D, Taulbut M, Hanlon P (2010). The aftershock of deindustrialization–trends in mortality in Scotland and other parts of post-industrial Europe.. European Journal of Public Health.

[6] McLoone P (2003). Increasing mortality among adults in Scotland 1981 to 1999.. European Journal of Public Health.

[7] Carstairs V, Morris R (1989). Deprivation: explaining differences in mortality between Scotland and England and Wales.. BMJ.

[8] Hanlon P, Lawder R, Buchanan D, Redpath A, Walsh D (2005). Why is mortality higher in Scotland than in England & Wales? Decreasing influence of socioeconomic deprivation between 1981 and 2001 supports the existence of a ‘Scottish effect’.. Journal of Public Health.

[9] Walsh D, Bendel N, Jones R, Hanlon P (2010). It’s not ‘just deprivation’: why do equally deprived UK cities experience different health outcomes?. Public Health.

[10] Mitchell R, Fowkes G, Blane D, Bartley M (2005). High rates of ischaemic heart disease in Scotland are not explained by conventional risk factors.. Journal of Epidemiology and Community Health.

[11] Norman P, Boyle P, Exeter D, Feng Z, Popham F (2011). Rising premature mortality in the Uk’s persistently deprived areas: only a Scottish phenomenon?. Social Science and Medicine.

[12] Reid D, Brett G, Hamilton P, Jarrett R, Keen H (1974). Cardiorespiratory disease and diabetes among middle-aged male civil servants.. Lancet i.

[13] Davey-Smith G, Hart C, Hole D, MacKinnon P, Gillis C (1998). Education and occupational social class: which is the more important indicator of mortality risk?. Journal of Epidemiology and Community Health.

[14] Hawthorne V, Watt G, Hart C, Hole D, Davey-Smith G (1995). Cardiorespiratory disease in men and women in urban Scotland: baseline characteristics of the Renfrew/Paisley (Midspan) population study.. Scottish Medical Journal.

[15] Hart C, MacKinnon P, Watt G, Upton M, McConnachie A (2005). The Midspan Studies. International Journal of Epidemiology.. International Journal of Epidemiology.

[16] (1966). General Register Office. Classification of Occupations 1966.. London: HMSO.

[17] Rose G, Blackburn H, Gillum R, Prineas R (1982). Cardiovascular survey methods.. Geneva: World Health Organisation.

[18] Council MR (1965). Definition and classification of chronic bronchitis for epidemiological purposes.. Lancet.

[19] Rose G, Blackburn H (1968). Cardiovascular survey methods.. Geneva: World Health Organisation.

[20] Kivimäki M, Ferrie J, Batty G, Davey-Smith G, Elovainio M (2008). Optimal form of operationalizing BMI in relation to all-cause and cause specific mortality.. Obesity.

[21] Smith G, Shipley M, Hole D, Hart C, Watt G (1995). Explaining male mortality differentials between the west of Scotland and the south of England.. Journal of Epidemiology and Community Health.

[22] Menvielle G (2010). Occupational exposures contribute to educational inequalities in lung cancer incidence among men: Evidence from the EPIC prospective cohort study.. International Journal of Cancer.

[23] Smith G, Hart C, Watt G, Hole D, Hawthorne V (1998). Individual social class, area-based deprivation, cardiovascular disease risk factors, and mortality: the Renfrew and Paisley Study.. Journal of Epidemiology and Community Health.

[24] Rose G, Shipley M (1986). Plasma cholesterol concentration and death from coronary heart disease: 10 year results of the Whitehall study.. BMJ.

[25] Morris R, Whincup P, Lampe F, Walker M, Wannamethee S (2001). Geographic variation in incidence of coronary heart disease in Britain: the contribution of established risk factors.. Heart.

[26] Leyland A, Dundas R, McLoone P, Boddy F (2007). Cause-specific inequalities in mortality in Scotland: two decades of change. A population-based study.. BMC Public Health.

[27] Dorling D, Rigby J, Wheeler B, Ballas D, Thomas B (2007). Poverty, wealth and place in Britain, 1968–2005.. Bristol: Policy Press and Joseph Rowntree Foundation.

[28] McCartney G, Collins C, Walsh D, Batty G (2011). Explaining Scotland’s mortality: towards a synthesis.. Glasgow: Glasgow Centre for Population Health.

[29] McCarttney G, Collins C, Walsh D, Batty G (2012). Explaining Scotland’s mortality: towards a synthesis.. Public Health.

[30] Smith G, Dorling D, Gordon D, Shaw M (1999). The widening health gap: what are the solutions?. Critical Public Health.

[31] Dorling D (2010). Injustice: why social inequality persists.. Bristol: Policy Press.

