# Supplementary material for: Why Do Males in Scotland Die Younger than Those in England? Evidence from Three Prospective Cohort Studies
Source: PLoS One. 2012 Jul 11;7(7):e38860. doi: 10.1371/journal.pone.0038860 (PMC3394776; doi:10.1371/journal.pone.0038860)
Supplement: Table S1 — Sensitivity analyses showing hazard ratios for mortality in the Scottish cohorts compared to the Whitehall Study+ (reference) when Whitehall heights are reduced by half an inch (rather than one inch). (DOC) [file pone.0038860.s001.doc]

Table S1 - Sensitivity analyses showing hazard ratios for mortality in the Scottish cohorts compared to the Whitehall Study^+^ (reference) when Whitehall heights are reduced by half an inch (rather than one inch)

| **Adjusted for** | | **All-cause mortality** | | **CHD mortality** | | **Stroke mortality** | | **Respiratory mortality** | | **Lung cancer mortality** | | **Mortality due to accidents and suicide** | | **Alcohol-related mortality** | |
| --- | --- | --- | --- | --- | --- | --- | --- | --- | --- | --- | --- | --- | --- | --- | --- |
|  |  | **Collaborative** | **Renfrew & Paisley** | **Collaborative** | **Renfrew & Paisley** | **Collaborative** | **Renfrew & Paisley** | **Collaborative** | **Renfrew & Paisley** | **Collaborative** | **Renfrew & Paisley** | **Collaborative** | **Renfrew & Paisley** | **Collaborative** | **Renfrew & Paisley** |
|  |  | **HR**  **(95% CI)** | **HR**  **(95% CI)** | **HR**  **(95% CI)** | **HR**  **(95% CI)** | **HR**  **(95% CI)** | **HR**  **(95% CI)** | **HR**  **(95% CI)** | **HR**  **(95% CI)** | **HR**  **(95% CI)** | **HR**  **(95% CI)** | **HR**  **(95% CI)** | **HR**  **(95% CI)** | **HR**  **(95% CI)** | **HR**  **(95% CI)** |
| Age | | 1.25  (1.21,1.30) | 1.41 (1.36,1.45) | 1.32  (1.23,1.41) | 1.41 (1.33,1.50) | 1.55  (1.37,1.75) | 1.73 (1.57,1.92) | 1.05  (0.93,1.18) | 1.17 (1.07,1.29) | 1.65  (1.45,1.89) | 1.98 (1.78,2.21) | 1.77  (1.33,2.36) | 2.00 (1.56,2.55) | 1.73  (1.44,2.09) | 2.28 (1.95,2.67) |
| Age, height | | 1.23  (1.18,1.28) | 1.36 (1.31,1.40) | 1.28  (1.19,1.37) | 1.33 (1.25,1.41) | 1.51  (1.34,1.71) | 1.65 (1.48,1.83) | 1.01  (0.90,1.14) | 1.09 (0.99,1.21) | 1.63  (1.43,1.87) | 1.94 (1.73,2.17) | 1.71  (1.28,2.28) | 1.86 (1.44,2.39) | 1.67  (1.38,2.01) | 2.11 (1.79,2.48) |
| Age, body mass index | | 1.25  (1.20,1.30) | 1.39 (1.35,1.44) | 1.31  (1.22,1.41) | 1.36 (1.28,1.44) | 1.54  (1.36,1.74) | 1.69 (1.53,1.87) | 1.04  (0.93,1.18) | 1.22 (1.11,1.34) | 1.65  (1.45,1.89) | 2.08 (1.87,2.32) | 1.77  (1.33,2.35) | 2.06 (1.61,2.63) | 1.73  (1.43,2.09) | 2.27 (1.94,2.65) |
| Multiply^±^ adjusted without socio-economic position | | 1.10  (1.05,1.14) | 1.07 (1.03,1.11) | 1.15  (1.07,1.23) | 1.00 (0.94,1.07) | 1.44  (1.27,1.63) | 1.35 (1.21,1.51) | 0.78  (0.69,0.88) | 0.73 (0.65,0.81) | 1.27  (1.11,1.46) | 1.42 (1.26,1.60) | 1.58  (1.18,2.11) | 1.79 (1.36,2.34) | 1.55  (1.27,1.87) | 1.86 (1.56,2.21) |
| Multiply^±^ adjusted including socio-economic position | | 1.03  (0.99,1.08) | 1.00 (0.96,1.04) | 1.10  (1.02,1.18) | 0.95 (0.88,1.02) | 1.45  (1.27,1.65) | 1.36 (1.20,1.54) | 0.72  (0.63,0.82) | 0.67 (0.60,0.76) | 1.09  (0.95,1.27) | 1.20 (1.05,1.37) | 1.52  (1.11,2.08) | 1.70 (1.25,2.31) | 1.45  (1.18,1.78) | 1.72 (1.41,2.10) |

+ Analyses for all-cause mortality are based on 13,884,3956 and 6813 men in the Whitehall, Collaborative and Renfrew & Paisley studies respectively. For cause specific mortality the number of men in the analyses are 13850, 3948 and 6792 respectively. The number of deaths is given in Table 2.

* Angina, ECG abnormality, respiratory symptoms or breathlessness.

± Multiply adjusted for :- age, smoking, FEV_1_, cardio-respiratory symptoms or history, height, systolic blood pressure, cholesterol, body mass index
