# Supplementary material for: Why Do Males in Scotland Die Younger than Those in England? Evidence from Three Prospective Cohort Studies
Source: PLoS One. 2012 Jul 11;7(7):e38860. doi: 10.1371/journal.pone.0038860 (PMC3394776; doi:10.1371/journal.pone.0038860)
Supplement: Table S2 — Sensitivity analyses showing hazard ratios for all-cause mortality in the Scottish cohorts compared to the Whitehall Study+ (reference) when Whitehall heights are reduced by half an inch (rather than one inch). (DOC) [file pone.0038860.s002.doc]

Table S2 - Sensitivity analyses showing hazard ratios for all-cause mortality in the Scottish cohorts compared to the Whitehall Study^+^ (reference) when Whitehall heights are reduced by half an inch (rather than one inch)

|  | **Social classes I & II** | | | **Social class III NM** | | | **Social classes IIIM, IV, V** | | |
| --- | --- | --- | --- | --- | --- | --- | --- | --- | --- |
|  | **Whitehall I** | **Collaborative** | **Renfrew & Paisley** | **Whitehall I** | **Collaborative** | **Renfrew**  **& Paisley** | **Whitehall I** | **Collaborative** | **Renfrew**  **& Paisley** |
| **Number of subjects** | 10114 | 1220 | 1297 | 2333 | 664 | 804 | 1437 | 2072 | 4712 |
| **Number of deaths** | 8251 | 923 | 1004 | 2097 | 560 | 681 | 1338 | 1759 | 4026 |
| **Age, height** | 1.0 | 1.09  (1.02,1.17) | 1.21  (1.13,1.29) | 1.0 | 1.03  (0.93,1.13) | 1.11  (1.01,1.21) | 1.0 | 1.03  (0.96,1.11) | 1.10  (1.03,1.17) |
| **Age, body mass index** | 1.0 | 1.09  (1.02,1.17) | 1.19  (1.12,1.28) | 1.0 | 1.02  (0.93,1.12) | 1.10  (1.01,1.20) | 1.0 | 1.07  (0.99,1.15) | 1.14  (1.07,1.22) |
| **Multiply^±^ adjusted** | 1.0 | 1.01  (0.94,1.08) | 1.00  (0.93,1.07) | 1.0 | 0.97  (0.88,1.07) | 0.95  (0.87,1.05) | 1.0 | 1.05  (0.97,1.13) | 1.02  (0.95,1.09) |

* Angina, ECG abnormality, respiratory symptoms or breathlessness.

± Multiply adjusted for :- age, smoking, FEV_1_, cardio-respiratory symptoms or history, height, systolic blood pressure, cholesterol, body mass index
